# Exploring the hemoglobin-to-red blood cell distribution width ratio (HRR) to peripheral arterial disease nexus: a comprehensive analysis of NHANES data from 1999 to 2004

**DOI:** 10.3389/fphar.2025.1529155

**Published:** 2025-01-22

**Authors:** Zhihai Yu, Bin Lu, Rui Han, Can Tu

**Affiliations:** Interventional Department, The First Affiliated Hospital of Ningbo University, Ningbo, Zhejiang, China

**Keywords:** peripheral artery disease, hemoglobin-to-red blood cell distribution width ratio, ankle-brachial index, inflammation, epidemiology

## Abstract

**Objective:**

This study aimed to investigate the correlation between the Hemoglobin-to-Red Blood Cell Distribution Width Ratio (HRR) and Peripheral Artery Disease (PAD) prevalence, utilizing data from the National Health and Nutrition Examination Survey (NHANES) between 1999 and 2004.

**Methods:**

The study employed a cross-sectional design, analyzing data from 5,196 participants aged 40 and above. PAD was diagnosed using the Ankle-Brachial Index (ABI), with ABI less than 0.9 indicating PAD. HRR, calculated as the ratio of hemoglobin (HB) to red blood cell distribution width (RDW), was stratified into quartiles. Covariates included demographic and clinical variables such as BMI, lipid profiles, and diabetes status. Logistic regression analysis was conducted to assess the relationship between HRR and PAD, adjusting for potential confounders.

**Results:**

The study found that higher HRR quartiles were associated with a decreased risk of PAD. After adjusting for confounders, the odds ratios for PAD in relation to the second, third, and fourth quartiles of HRR compared to the first quartile were 0.71, 0.62, and 0.44, respectively (P < 0.001). A one-unit increase in HRR corresponded to a 56% reduction in the probability of PAD. ROC analysis indicated HRR as a stronger protective factor for PAD compared to other variables. Stratified analyses revealed that younger age and lower BMI amplified the protective effect of HRR on PAD.

**Conclusion:**

The study demonstrated a significant inverse relationship between HRR and PAD, suggesting that HRR may serve as a protective factor against PAD. This finding highlights the potential role of HRR in the pathogenesis of PAD and its clinical implications.

## Introduction

Peripheral Artery Disease (PAD) typically refers to a group of conditions characterized by arterial narrowing or occlusion in the lower extremities, excluding the coronary arteries, aorta, and intracranial segments (Bevan and White Solaru, 2020; Mehta et al., 2021; Tran, 2021). The diagnosis of PAD can be made by measuring the blood pressure in the posterior tibial artery and the brachial artery, which involves calculating the Ankle-Brachial Index (ABI), defined as the systolic pressure in the posterior tibial artery divided by the systolic pressure in the brachial artery (Makowsky et al., 2008; Conen et al., 2009; Barnes et al., 2020). PAD is diagnosed when the ABI is less than 0.9 (Hicks et al., 2023; Mandaglio-Collados et al., 2023). It is reported that over 200 million people worldwide suffer from PAD, with the prevalence increasing with age and affecting a significant proportion of the elderly population (Shu and Santulli, 2018; Mastracci et al., 2022). Individuals with PAD mainly exhibit symptoms such as intermittent claudication, rest pain, abnormal skin temperature, discoloration, and sensory abnormalities, with severe cases potentially leading to non-traumatic amputations and even death (Geiss et al., 2019; Mahe et al., 2022). This not only impacts the health of the elderly but also increases the disability rate in the population, imposing a substantial economic burden on families and society (Matsushita et al., 2022). Therefore, PAD is not just a health issue; it also involves public health challenges related to family happiness, economic prosperity, and social harmony. The etiology of PAD is diverse, including hypertension (Agnelli et al., 2020), diabetes (Krzyzanowska et al., 2006), hyperlipidemia (Selvaggio et al., 2020), obesity, inflammation (Chan et al., 2022), and lifestyle factors (Criqui et al., 2021) Among these, inflammation is a particularly intriguing area of research with implications for targeted treatment (Brevetti et al., 2010; Alnima et al., 2023).

HRR is recognized as an inflammatory marker associated with the incidence and adverse events of various diseases (Qu et al., 2021), such as diabetes (Wang et al., 2020; Xiu et al., 2022), non-small cell lung cancer (Chen et al., 2020), and atrial fibrillation (Wang et al., 2022). HRR is primarily composed of two hematological indicators: hemoglobin (HB) and red blood cell distribution width (RDW) (Zhao et al., 2022; Song et al., 2023). HB mainly reflects the degree of anemia (Gell, 2018), while RDW indicates the heterogeneity in red blood cell volume and is indirectly used for diagnosing anemia (Fava et al., 2019; Yang et al., 2023). Previous studies (Li et al., 2021; Guaní-Guerra et al., 2022) have shown that HRR undergoes dynamic changes in response to disease stimuli, and its level variations can serve as a sensitive measure of inflammation (Nan et al., 2024). Although HRR, as an inflammatory marker, is related to numerous diseases, the clinical relationship between HRR and PAD remains unclear. PAD is a disease associated with inflammation and immunity, while HRR is an indicator that reflects the levels of inflammation and immune metabolism in the body. Therefore, exploring the role of HRR in PAD is of great importance.

Given that PAD is a condition that may be associated with inflammation, there might be a unique link between HRR and PAD. However, the relationship between HRR and PAD is not clear in current research. Therefore, in this study, we extracted data on HRR and PAD from the National Health and Nutrition Examination Survey (NHANES) between 1999 and 2004, attempting to explore whether there is a specific correlation between HRR and the prevalence of PAD.

## Materials and methods

### Study population and study design

**FIGURE 1:**
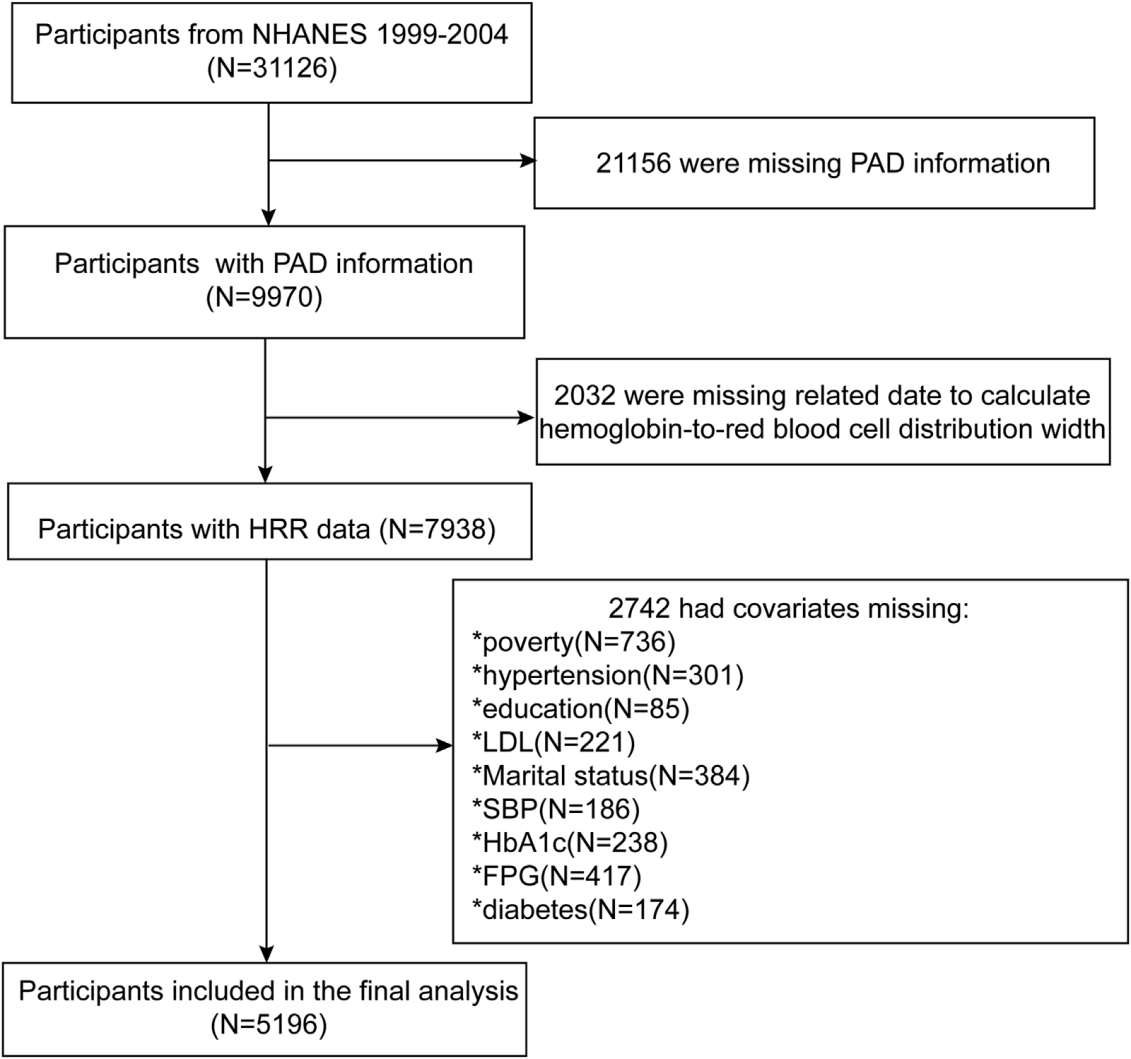
Research flowchart.

The NHANES is a cross-sectional, nationally representative survey of the non-institutionalized civilian population in the United States (US). It includes interviews and standardized physical examinations, with a focus on collecting data through anthropometric measurements, blood samples, and self-report questionnaires. Additionally, at the outset of the NHANES, informed consent was obtained from each participant. This obviates the need for further provision of ethical clearance for this study. Figure 1 provides a detailed depiction of the participant selection process for this study (Figure 1). Initially, we extracted demographic, laboratory, physical examination, and questionnaire data from a total of 31,126 participants in the NHANES spanning from 1999 to 2004. Subsequently, since NHANES only included PAD-related examinations for individuals aged 40 and above, the first step was to exclude those lacking PAD-related information, resulting in the exclusion of 21,156 participants. Next, we removed 2,032 participants due to missing data required for the calculation of the HRR. To further enhance the credibility of the study, we proceeded to screen for potential confounding factors, leading to the exclusion of an additional 2,742 participants who had missing information regarding these factors. Specifically, 736 individuals were missing income level Poverty Impact Ratio (PIR) data, 301 lacked hypertension-related data, 85 had unknown education levels, 221 were without low-density lipoprotein (LDL) data, 384 did not provide marital status information, 186 had not had their systolic blood pressure measured, 238 lacked glycated hemoglobin data, 417 were missing fasting glucose data, and another 174 had no diagnosed diabetes-related information. After these exclusions, a final total of 5,196 participants were included in the study.

### Measurement of ABI and PAD

Leveraging the examination data component of NHANES, one of its four main components, we assessed the ankle brachial blood pressure index (ABI) as a measure for evaluating lower extremity disease. Specifically, we measured the systolic blood pressure of the right brachial artery and the posterior tibial artery bilaterally at the ankle joints of the participants. In cases where participants’ right arms were not accessible for measurement, the left brachial artery systolic blood pressure was used as a substitute for the right brachial artery systolic blood pressure. Given that NHANES collected this data with varying measurement frequencies for different age groups, the calculation of ABI was stratified by age. 1) For individuals between the ages of 40–59, the ABI was calculated by dividing the average of two measurements of the posterior tibial artery systolic blood pressure bilaterally by the average of two brachial artery systolic blood pressure measurements. 2) For those aged 60 and above, who had only one measurement of the brachial artery systolic blood pressure, the ABI was determined by dividing the average of two measurements of the posterior tibial artery systolic blood pressure bilaterally by a single brachial artery systolic blood pressure measurement. According to previous studies, participants with an ABI less than 0.9 were defined as having PAD.

### Definition of the HRR

The hemoglobin-to-red blood cell distribution width ratio (HRR) is calculated by dividing hemoglobin (HB) levels by the red blood cell distribution width (RDW), utilizing laboratory data from the NHANES. For subsequent analysis, HRR is stratified into quartiles.

### Covariates

Building on previous related research, this study included waist circumference (WC), high-density lipoprotein (HDL), triglycerides (TG), low-density lipoprotein (LDL), total cholesterol (TC), age, poverty-income ratio (PIR), body mass index (BMI), systolic blood pressure (SBP), glycated hemoglobin (HbA1C), fasting plasma glucose (FPG), gender, marital status, race, education level, hypertension, and diabetes as covariates.

BMI is calculated from anthropometric data, specifically height (HT in meters, m) and weight (WT in kilograms, kg), using the formula BMI = WT/(HT) ^ ^2^.

Marital status is categorized into three main groups: 1) Married/cohabiting, 2) Widow/widower/separated, and 3) Unmarried.

Race is classified into five categories based on NHANES survey results: Mexican, Hispanic, White, Black, and Other Race.

Education level is primarily divided into three categories: Under high school, High school, and Over high school.

Hypertension is defined by three main criteria: 1) Systolic blood pressure of 140 mmHg or higher in anthropometric data, 2) Diastolic blood pressure of 90 mmHg or higher in anthropometric data, and 3) Participants who have been told by a doctor that they have hypertension, as reported in the blood pressure questionnaire. Meeting any one of these criteria is sufficient to be defined as having hypertension.

Diabetes is defined by four criteria: 1) FPG of 7 mmol/L or higher, 2) HbA1C of 6.5% or higher, 3) Participants who have been told by a doctor that they have diabetes, as reported in the questionnaire, and 4) Participants who have taken hypoglycemic medications, as reported in the questionnaire. Meeting any one of these criteria is sufficient to be defined as having diabetes.

### Statistical analysis

In this study, we conducted a thorough processing and quality control of the data. Given the non-normal distribution of the data, continuous variables are expressed as medians with interquartile ranges (Q1, Q3), while categorical variables are presented as counts and percentages. Comparisons between the PAD and non-PAD groups were made using the Kruskal-Wallis test for continuous variables and the chi-squared test for categorical variables. A p-value less than 0.05 is considered to indicate a statistically significant difference.

Utilizing logistic regression analysis appropriate for the survey design, we explored the relationship between HRR and PAD. The robustness of the model was incrementally enhanced by adjusting for different covariates. Specifically, Model 1 is the most basic model, with no adjustments for any variables. Model 2 is adjusted for race, age, and gender. Finally, Model 3 includes adjustments for potential confounding factors such as gender, age, PIR, education level, race, BMI, HDL, TG, LDL, TC, SBP, HbA1C, FPG, WC, hypertension, and diabetes to further strengthen the robustness of the model.

## Results

### Demographic and initial characteristics of participants

The study selected a cohort of 5,196 adult Americans from the NHANES database spanning from 1999 to 2004, with a higher number of males, totaling 2,684 individuals, accounting for approximately 51.66% of the study population. Within this cohort, 316 individuals were diagnosed with PAD, representing 6.08% of the sample size. The majority of participants were identified as non-Hispanic whites, with 2,890 individuals making up 55.62% of the total study cohort. Table 1 summarizes the demographic and baseline characteristics of the study subjects, stratified by HRR quartiles (Table 1). Individuals with higher HRR tended to be younger (P < 0.001), have a higher proportion of males (P < 0.001), and were free from hypertension (P < 0.001). Additionally, this group was likely to exhibit lower HDL levels (P < 0.001) and higher TG levels in their blood biochemistry (P < 0.001). However, there were no significant differences in fasting plasma glucose (FPG) (P = 0.223) and diabetes (P = 0.163) across HRR quartiles.

**TABLE 1:** Characteristics of the participants categorized by HRR.

HRR	Q1 (0.248–1.041)	Q2 (1.042–1.143)	Q3 (1.144–1.231)	Q4 (1.231–1.597)	*P*-value
WC, cm	97.20 (62.00–157.80)	97.50 (59.65–156.60)	98.00 (61.20–152.70)	99.10 (60.20–157.10)	<0.001
HDL, mmol/L	1.37 (0.21–3.98)	1.37 (0.31–3.15)	1.29 (0.49–3.03)	1.18 (0.49–4.94)	<0.001
TG, mmol/L	1.25 (0.29–25.70)	1.43 (0.29–27.06)	1.49 (0.27–10.35)	1.65 (0.36–43.51)	<0.001
LDL, mmol/L	3.21 (0.34–5.95)	3.21 (0.93–6.10)	3.21 (0.54–7.16)	3.21 (1.03–9.34)	<0.001
TC, mmol/L	5.15 (2.20–12.41)	5.35 (2.87–11.04)	5.35 (2.90–11.02)	5.35 (2.48–12.00)	<0.001
Age, years	64.00 (40.00–85.00)	63.00 (40.00–85.00)	59.00 (40.00–85.00)	55.00 (40.00–85.00)	<0.001
PIR	2.09 (0.00–6.07)	2.35 (0.00–6.71)	2.55 (0.00–7.36)	2.51 (0.00–7.98)	<0.001
BMI, kg/m2	27.46 (16.16–62.47)	27.41 (15.80–57.20)	27.40 (15.30–55.40)	27.37 (14.70–51.04)	0.005
SBP, mmHg	131.00 (84.00–230.00)	131.00 (64.00–228.00)	131.00 (80.00–230.00)	130.00 (86.00–212.00)	<0.001
HBA1C, %	5.50 (3.90–14.30)	5.50 (3.90–18.80)	5.40 (3.90–14.00)	5.40 (4.20–18.00)	<0.001
FPG, mmol/L	6.15 (2.88–22.07)	6.15 (2.13–32.31)	6.15 (2.54–23.09)	6.15 (3.14–30.40)	0.223
RDW, %	13.60 (11.60–30.50)	12.70 (11.10–15.90)	12.50 (11.00–15.90)	12.20 (10.60–14.30)	<0.001
HB, g/dL	12.80 (6.20–16.30)	14.00 (12.10–17.40)	14.80 (12.60–19.20)	15.90 (13.80–19.70)	<0.001
ABI	1.09 (0.41–2.11)	1.11 (0.38–2.09)	1.12 (0.36–1.87)	1.14 (0.55–2.10)	<0.001
Gender, n (%)					<0.001
male	396 (30.51)	488 (37.63)	749 (57.93)	1051 (80.35)	
female	902 (69.49)	809 (62.37)	544 (42.07)	257 (19.65)	
Marital status, n (%)					<0.001
Married/cohabiting	712 (54.85)	789 (60.83)	874 (67.59)	937 (71.64)	
Widow/widower/separated	474 (36.52)	402 (30.99)	296 (22.89)	253 (19.34)	
Unmarried	112 (8.63)	106 (8.17)	123 (9.51)	118 (9.02)	
Race, n (%)					<0.001
Mexican	227 (17.49)	261 (20.12)	270 (20.88)	329 (25.15)	
Hispanic	42 (3.24)	57 (4.39)	46 (3.56)	47 (3.59)	
White	554 (42.68)	721 (55.59)	802 (62.03)	813 (62.16)	
Black	424 (32.67)	224 (17.27)	142 (10.98)	73 (5.58)	
Other Race	51 (3.93)	34 (2.62)	33 (2.55)	46 (3.52)	
Education, n (%)					0.003
Under high school	501 (38.60)	437 (33.69)	431 (33.33)	410 (31.35)	
High school	323 (24.88)	322 (24.83)	313 (24.21)	330 (25.23)	
Over high school	474 (36.52)	538 (41.48)	549 (42.46)	568 (43.43)	
Hypertension, n (%)					<0.001
No	876 (67.49)	859 (66.23)	925 (71.54)	948 (72.48)	
Yes	422 (32.51)	438 (33.77)	368 (28.46)	360 (27.52)	
Diabetes, n (%)					0.163
No	1086 (83.67)	1102 (84.97)	1105 (85.46)	1135 (86.77)	
Yes	212 (16.33)	195 (15.03)	188 (14.54)	173 (13.23)	
PAD, n (%)					<0.001
No	1171 (90.22)	1208 (93.14)	1228 (94.97)	1272 (97.25)	
Yes	127 (9.78)	89 (6.86)	65 (5.03)	36 (2.75)	

Data were expressed as n (%) and median (interquartile range).

HRR, hemoglobin-to-red blood cell distribution width ratio, WC, waist circumference; HDL, high-density lipoprotein; TG, triglycerides; LDL, low density lipoprotein; TC, total cholesterol; FPG, fasting plasma glucose; ABI, ankle brachial index; PIR, poverty income ratio; BMI, body mass index; SBP, systolic blood pressure; HB, hemoglobin; RDW, red blood cell distribution width; PAD peripheral arterial disease.

### Univariate logistic analyses revealed the associations between multiple variables with PAD

As shown in Table 2, in the univariate analysis, HB (OR = 0.84, 95%CI: 0.78–0.90), and HRR (OR = 0.08, 95%CI: 0.04–0.15) were negatively associated with PAD (Table 2).

**TABLE 2:** Weighted univariate logistic analyses between Variables and PAD (odds ratios, 95% confidence intervals).

Variables	Univariate analysis (crude model)	
	OR95% CI	*P*-value
WC	1.00 (0.99, 1.01)	0.8567
BMI	0.98 (0.96, 1.00)	0.0420
TG	1.01 (0.94, 1.08)	0.8772
LDL	1.04 (0.87, 1.25)	0.6684
TC	0.97 (0.87, 1.08)	0.6143
SBP	1.02 (1.02, 1.03)	<0.0001
HB	0.84 (0.78, 0.90)	<0.0001
RDW	1.27 (1.19, 1.36)	<0.0001
HRR	0.08 (0.04, 0.15)	<0.0001

### Receiver operating characteristic (ROC) analysis revealed HRR shows relatively better predictive performance

**FIGURE 2:**
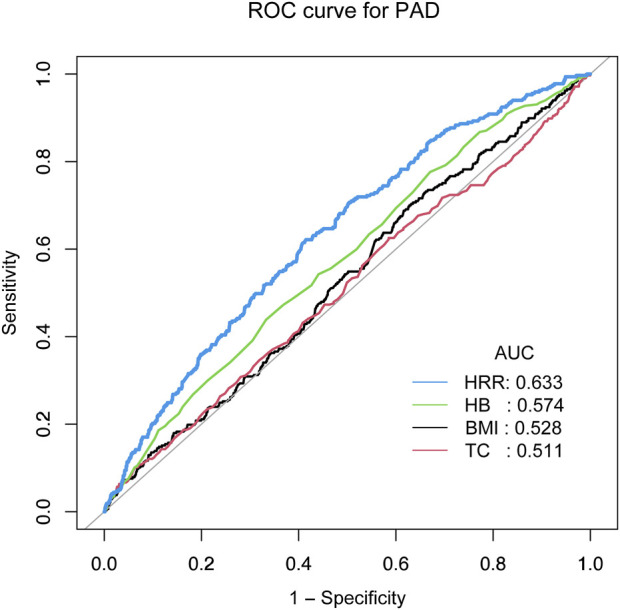
ROC analysis revealed HRR shows relatively better predictive performance.

To further elucidate the impact of the aforementioned factors on PAD, we conducted a ROC curve analysis. The ROC curves indicate that compared to HB (AUC = 0.574), BMI (AUC = 0.528), and TC (AUC = 0.511), HRR shows relatively better predictive performance. (AUC = 0.633) (Figure 2).

### The relationship between PAD and HRR quartiles

Referencing Table 3, elevated HRR quartiles are linked to a decreased risk of PAD (Table 3). The unadjusted model reveals an inverse relationship between HRR quartiles and PAD prevalence (Model 1). This correlation retains its significance following partial adjustments in Model 2. With a comprehensive consideration of all potential confounding factors, the OR for PAD in relation to the second (Q2), third (Q3), and fourth (Q4) quartiles compared to the reference quartile (Q1) are approximately 0.71 (95% CI: 0.59–0.86), 0.62 (95% CI: 0.53–0.73), and 0.44 (95% CI: 0.29–0.68), respectively, for Model 3 (P < 0.001). Additionally, After adjusting for factors such as gender, age, PIR, education level, race, BMI, HDL, TG, LDL, TC, SBP, HbA1C, FPG, WC, hypertension, and diabetes, an increase of one unit in HRR corresponds to a 56% reduction in the probability of PAD, as demonstrated by the OR.

**TABLE 3:** Multivariate Cox regression analysis of HRR with PAD.

	HRR quartiles				*P* for trend
	Q1	Q2	Q3	Q4	
	0.248–1.041	1.042–1.143	1.144–1.231	1.231–1.597	
	OR (95%CI)	OR (95%CI)	OR (95%CI)	OR (95%CI)	
PAD	127 (9.78%)	89 (6.86%)	65 (5.03%)	36 (2.75%)	
Model 1	Reference	0.68 (0.51, 0.90)	0.49 (0.36, 0.67)	0.26 (0.18, 0.38)	<0.001
Model 2	Reference	0.74 (0.60, 0.92)	0.67 (0.54, 0.83)	0.49 (0.32, 0.74)	<0.001
Model 3	Reference	0.71 (0.59, 0.86)	0.62 (0.53, 0.73)	0.44 (0.29, 0.68)	<0.001

Values are presented as weighted odds ratios (ORs), 95% confidence interval (95%CI), and *P* value.

Model 1 adjusted for none.

Model 2 adjusted for gender, age and race.

Model 3 adjusted for gender, age, PIR, education level, race, BMI, HDL, TG, LDL, TC, SBP, HBA1C, FPG, WC, hypertension and diabetes.

**FIGURE 3:**
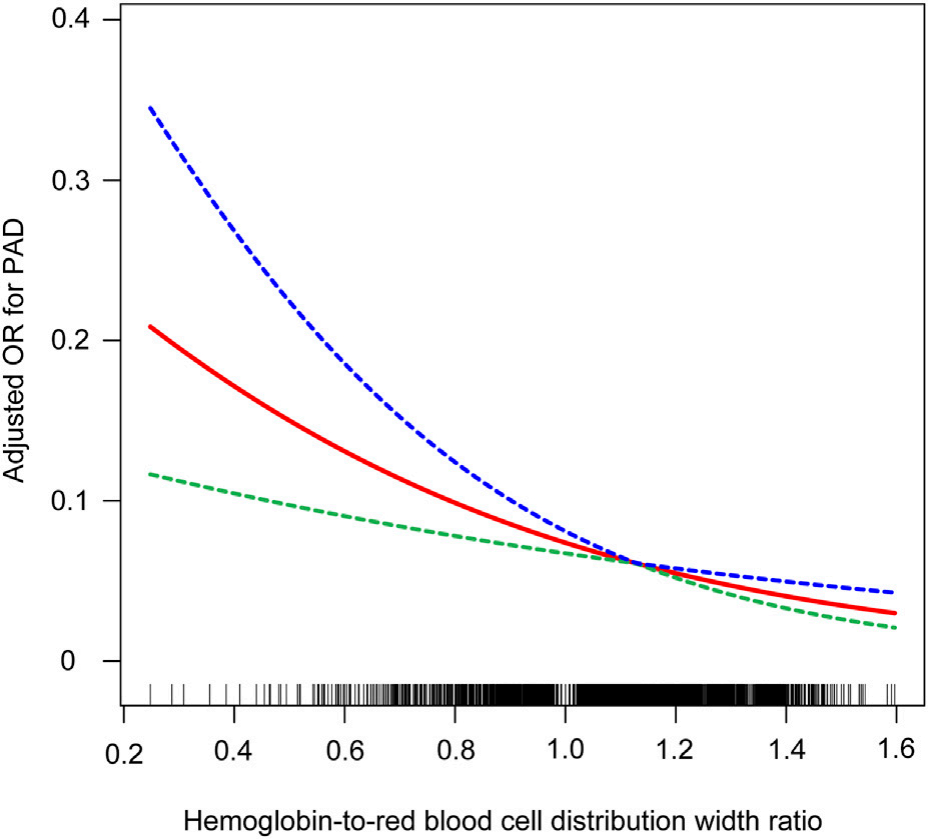
Relationship between HRR and PAD. the blue line represents the upper limit of the 95% confidence interval for the adjusted OR values, the green line represents the lower limit of the 95% confidence interval for the adjusted OR values, and the red line represents the adjusted OR values themselves.

Utilizing smooth curve fitting and threshold effect analysis, we assessed the potential linear correlation between HRR and the risk of PAD. As shown in Figure 3, the findings indicated a substantial linear association between these variables (Figure 3). Table 4 details the outcomes from a standard linear regression model, revealing an 80% reduction in PAD risk for each one-unit increment in HRR (P < 0.0001). Two-piecewise linear regression models identified a critical point at an HRR value of 1.12. For HRR values at or below 1.12, an increment of one unit in HRR is associated with a 75% decrease in PAD odds (OR = 0.25, 95%CI = 0.10–0.64; P < 0.0036). Beyond an HRR of 1.12, an additional unit increase in HRR is linked to a 96% decrease in PAD odds (OR = 0.04, 95%CI = 0.00–1.54; P = 0.845), and the log-likelihood ratio test yields a P value of 0.364. Consequently, when synthesizing these findings, a definite linear relationship is observed between HRR and PAD risk.

**TABLE 4:** Threshold and saturation effect analysis of HRR on PAD.

Outcomes	PAD	
	OR (95%CIs)	P value
Model a		
(Fitting model by standard linear regression)	0.20 (0.09, 0.45)	<0.0001
Model b		
(Fitting models by two-piecewise linear regression)		
Infection point	1.12	
<Infection point	0.25 (0.10, 0.64)	0.0036
>Infection point	0.04 (0.00, 1.54)	0.0845
P for log-likelihood ratio test	0.364	

The two-piecewise regression models were adjusted for gender, age, PIR, education level, race, BMI, HDL, TG, LDL, TC, SBP, HBA1C, FPG, WC, hypertension and diabetes.

OR, odds ratios, 95% CI, 95% confidence interval.

### Stratified analysis

As indicated in Table 5, we conducted a series of stratified analyses to assess the robustness of the association between HRR and PAD and to uncover any potential differences among various demographic subgroups (Table 5). In the majority of these stratified subgroups, HRR emerged as a significant protective factor for PAD. Notably, as mentioned in Table 1, age has a substantial impact on PAD, and younger individuals tend to have higher HDL levels, which becomes evident in the stratified analysis. That is, the younger the population, the stronger the influence of HRR on PAD (P = 0.0002). Furthermore, regarding BMI, lower BMI values correspond to a reduced risk of PAD (P < 0.0001). These findings suggest that individuals who are obese, older, and have lower HRR values may be more susceptible to PAD.

**TABLE 5:** Stratified analysis of the correlation between HRR and PAD in adults in the NHANES 1999–2004.

Subgroup	PAD	
	OR (95%CI)	P-value
Age, years		
40–51	0.04 (0.01, 0.20)	0.0002
52–66	0.25 (0.05, 1.21)	0.0853
67–85	0.11 (0.05, 0.26)	<0.0001
PIR		
0–1.577	0.10 (0.04, 0.26)	<0.0001
1.58–3.483	0.11 (0.04, 0.29)	<0.0001
3.49–7.982	0.05 (0.01, 0.21)	<0.0001
SBP, mmHg		
64–120	0.05 (0.01, 0.22)	<0.0001
122–134	0.11 (0.04, 0.32)	<0.0001
136–230	0.08 (0.03, 0.20)	<0.0001
HBA1C, %		
3.9–5.25	0.10 (0.03, 0.39)	0.0009
5.3–5.65	0.07 (0.02, 0.20)	<0.0001
5.7–18.8	0.09 (0.03, 0.23)	<0.0001
TG, mmol/L		
0.271–1.1235	0.07 (0.03, 0.21)	<0.0001
1.129–1.705	0.04 (0.01, 0.17)	<0.0001
1.715–43.512	0.09 (0.04, 0.25)	<0.0001
HDL, mmol/L		
1.14–1.46	0.04 (0.01, 0.12)	<0.0001
1.47–4.94	0.11 (0.04, 0.32)	<0.0001
2.2–4.885	0.10 (0.03, 0.33)	0.0002
TC, mmol/L		
4.89–5.72	0.09 (0.03, 0.24)	<0.0001
5.74–12.41	0.09 (0.03, 0.25)	<0.0001
14.7–25.42	0.07 (0.02, 0.24)	<0.0001
BMI, kg/m2		
25.43–29.54	0.05 (0.02, 0.15)	<0.0001
29.55–62.47	0.06 (0.02, 0.17)	<0.0001
59.65–92.1	0.14 (0.04, 0.46)	0.0012

## Discussion

PAD is a syndrome of peripheral circulatory dysfunction, with the broad sense of peripheral arterial disease referring to atherosclerotic changes caused by large arteries other than the coronary arteries and aorta, while the narrow sense refers specifically to lower extremity arterial diseases. Among the many etiologies of PAD, inflammation plays an extremely important role. By exploring data from the NHANES between 1999 and 2004, our study reveals an approximate linear association between HRR and PAD. This indicates a significant negative correlation between HRR and the likelihood of PAD.

Inflammation is a key element in the pathophysiology of PAD (Fort-Gallifa et al., 2017), a condition associated with inflammation (Tantry et al., 2020). RDW (Hu et al., 2020) and HRR (Liu and Wang, 2023), both implicated in inflammation, are considered novel inflammatory biomarkers (Xi et al., 2024). Previous studies have shown RDW to be useful in predicting mortality rates among PAD patients. In a study examining 1,039 consecutive outpatients from January 1997 to December 2007, with follow-up through September 2009, the cohort had an average age of 69.5 ± 12.0 years, with approximately 60.9% males and 97.6% whites. After adjusting for age, gender, cardiovascular risk factors, and comorbidities, patients in the highest RDW quartile (>14.5%) had a 66% higher risk of death compared to those in the lowest quartile (<12.8%, p < 0.0001); a 1% increase in RDW was associated with a 10% increase in the risk of all-cause mortality (hazard ratio 1.10, 95% confidence interval 1.08 to 1.12, p < 0.0001). RDW was ultimately found to be an independently available prognostic indicator for PAD patients (Ye et al., 2011). Zalawadiya et al., studying 6,950 participants in the NHANES database from 1999 to 2004, found that higher levels of RDW were independently associated with a higher risk of PAD and significantly improved risk prediction beyond the estimates of the American College of Cardiology’s PAD screening criteria (Zalawadiya et al., 2012). Studies on HRR have yielded different conclusions. A study including 3,745 adult COPD patients from the NHANES database between 1999 and 2018 explored the association between RDW and HRR levels and mortality. After adjusting for various potential confounders, higher RDW levels were positively associated with all-cause mortality risk (HR = 1.16, 95% CI = 1.11–1.21, P < 0.001) and cardiovascular disease (CVD) mortality risk (HR = 1.13, 95% CI = 1.06–1.21, P < 0.001). HRR was negatively associated with all-cause mortality risk (HR = 0.14, 95% CI = 0.08–0.25, P < 0.001) and CVD mortality risk (HR = 0.12, 95% CI = 0.05–0.31, P < 0.001), concluding that higher RDW levels in COPD patients were positively correlated with increased mortality risk, while HRR levels were negatively correlated with all-cause and CVD mortality risks (Liu et al., 2024). Bozkaya et al., in a retrospective screening of nasopharyngeal carcinoma (LANC) patients treated with chemotherapy and radiotherapy between October 2010 and June 2020, found that in the low HRR group, overall survival (OS) and disease-free survival (DFS) were 44.4 months (95% CI: 4.9–83.8) and 15.7 months (95% CI: 0.1–36.2), respectively, but not attainable in the high HRR group (p < 0.001). They concluded that HRR is an independent prognostic marker for OS and DFS in LANC patients treated with chemotherapy and radiotherapy (Bozkaya et al., 2023). Studies have been conducted on the association between red blood cell distribution width (RDW) and the ratio of RDW to platelet count (RPR) with cardiovascular disease (CVD), and it has been found that the association between RDW and the prevalence of CVD is more pronounced in women and smokers (all interaction p-values <0.05). It was concluded that there is statistical heterogeneity in the association between RDW, RPR distribution, and the prevalence of CVD across gender, smoking status, and age groups (Veeranna et al., 2013; Shah et al., 2017; Ainiwaer et al., 2023). Therefore, HRR may play different roles in various diseases. This study addresses this controversial point by exploring the potential link between HRR and PAD using a large dataset from NHANES. Notably, this study found that high levels of HRR are a protective factor for PAD, with each unit increase in HRR reducing the probability of PAD by 56%. The results differ from those of Bozkaya et al., possibly because their study focused on oncology patients who underwent chemotherapy and radiotherapy, leading to varying degrees of change in hemoglobin levels. This is one possible explanation, and further research is needed to confirm it.

Under rigorous inclusion criteria, this study carefully selected study indicators and specific populations from the reputable NHANES database to ensure a certain level of persuasiveness in the results. We made every effort to adjust for a range of covariates to further ensure the reliability of the findings. However, it must be acknowledged that there are several limitations. The study is cross-sectional, meaning it can only explore potential associations between HRR and PAD, not causal relationships. Additionally, while this study controlled for multiple covariates, there may be other confounding factors that could affect the relationship between HRR and PAD, and it was not possible to include all potential confounders in this study. Despite the aforementioned limitations, this study is novel in being the first to explore the possible role of HRR in PAD and finds that as HRR levels increase, the risk of PAD decreases. This provides a foundation and new insights for future research.

## Conclusion

This study reveals a robust inverse relationship between the HRR and PAD, highlighting HRR as a significant protective factor against PAD. The findings underscore the importance of HRR in PAD risk assessment, particularly among demographic subgroups, and contribute to a deeper understanding of the role of inflammation in PAD pathogenesis.

## Data Availability

Publicly available datasets were analyzed in this study. This data can be found here: 1999–2000: https://wwwn.cdc.gov/nchs/nhanes/ContinuousNhanes/Default.aspx?BeginYear=1999 2001–2002: https://wwwn.cdc.gov/nchs/nhanes/ContinuousNhanes/Default.aspx?BeginYear=2001 2003–2004: https://wwwn.cdc.gov/nchs/nhanes/ContinuousNhanes/Default.aspx?BeginYear=2003.
